# Supplementary material for: Bta06987, Encoding a Peptide of the AKH/RPCH Family: A Role of Energy Mobilization in Bemisia tabaci
Source: Insects. 2022 Sep 13;13(9):834. doi: 10.3390/insects13090834 (PMC9502992; doi:10.3390/insects13090834)
Supplement: Supplementary file 1 [file insects-13-00834-s001.zip › insects-1856930-supplemental data Figures and table.pdf]

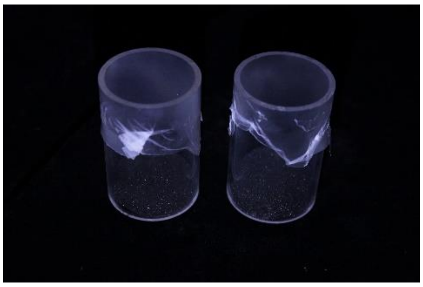

Figure S1. Device for starvation research and RNAi expertiment.

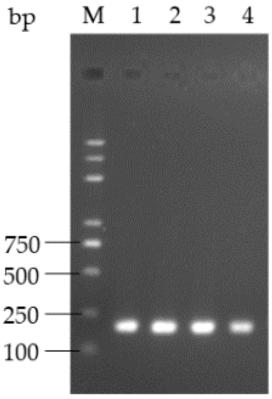

Figure S2.Detection of the *Bta06987*

atg act tgt cgc aca att ctg gtg tta ctc aca aat gca atc ctc ctg tcc gct ctg tgc
M T C R T I L V L L T N A I L L S A L C
tac gcc cag gtc aac ttc tcg ccc acg tgg ggc aag aga acc gtc acg caa gaa gaa tgc
Y A Q V N F S P T W G K R T V T Q E E C
acc tcc aag cct tcc atg gaa ctt ctc atg tat tta tac aag atg atc gag aat gag gcg
T S K P S M E L L M Y L Y K M I E N E A
caa aaa ata tca gat tgt gag aag ttc aga agt taa
Q K I S D C E K F R S -

Figure S3. DNA squences of *Bta06987* and predicted structures of neuropeptide precursor. Predicted signal peptides (highlighted in yellow), cleavage signals (red), putative bioactive mature peptides (light blue).

Table S1. The sequence of *Bta06987*

The sequence of *Bta06987*	ATGACTTGTCGCACAATTCTGGTGTTA<br>CTCACAAATGCAATCCTCCTGTCCGCT<br>CTGTGCTACGCCCAGGTCAACTTCTC<br>GCCCA<br>CGTGGGGCAAGAGAACCGTCACGCA<br>AGAAGAATGCACCTCCAAGCCTTCCA<br>TGGAACTTCTCATGTATTTATACAAGA<br>TGATCGA<br>GAATGAGGCGCAAAAAATATCAGATT<br>GTGAGAAGTTCAGAAGTTAA
